# Overview of tau biology as it relates to Alzheimer's pathophysiology, symptoms, and therapeutic development

**DOI:** 10.1002/alz70859_099838

**Published:** 2025-12-25

**Authors:** Lawren VandeVrede

**Affiliations:** ^1^ Memory and Aging Center, UCSF Weill Institute for Neurosciences, University of California San Francisco, San Francisco, CA USA

## Abstract

The protein tau is central to the pathophysiology of many neurodegenerative diseases, including Alzheimer’s disease (AD). Tau is ubiquitously expressed throughout the brain, and misfolded, insoluble tau protein deposits have been found at autopsy in neurons and glia in numerous neurodegenerative diseases, called tauopathies. In AD, a secondary tauopathy, aggregation of tau temporally follows amyloid plaque development. In AD, as in other tauopathies, the localization of tau corresponds with the resulting clinical features, and the amount of tau tangles seen on autopsy or positron emission tomography (PET) correlates with symptom severity throughout different stages of disease. As with amyloid, several lines of genetic evidence support tau’s role in neurodegeneration, particularly pathogenic variants in the MAPT gene that codes for tau, which result in a diverse spectrum of clinical presentations, collectively called primary tauopathies. The normal physiologic role of tau is likely manifold, but it is a soluble cytoplasmic protein that interacts with and potentially stabilizes microtubules through a conserved microtubule binding region. However, the proposed role of tau in neurodegenerative disease is thought to be a toxic gain of function, supported by genetic evidence from genome wide association studies (GWAS) that have only identified gain‐of‐function variants. Additional evidence is found in nonclinical animal models, including tau knockout mice that have little to no apparent phenotype. Pathogenic species of tau are likely driven and defined by specific post‐translational modifications, e.g., amyloid‐induced hyperphosphorylation in the case of AD. The specific mechanism of propagation after toxic species of tau are produced is through cell‐to‐cell spread after an initial “seeding” event. In several neurodegenerative diseases, electronic microscopy has revealed distinct tau conformations specific to different tauopathies, each of which may be a unique seed. Targeting this tau toxic gain‐of‐function is possible through numerous pharmacological approaches, and early evidence from clinical trials where tau is pharmacologically reduced has shown reduction to be generally safe and well‐tolerated.

